# Family history, socioeconomic factors, comorbidities, health behaviors, and the risk of sudden cardiac arrest

**DOI:** 10.1038/s41598-023-48357-1

**Published:** 2023-12-01

**Authors:** Eujene Jung, Jeong Ho Park, Young Sun Ro, Hyun Ho Ryu, Kyoung-Chul Cha, Sang Do Shin, Sung Oh Hwang, The Cardiac Arrest Pursuit Trial with Unique Registration, Epidemiologic Surveillance (CAPTURES) project investigators, Sung Oh Hwang, Sang Do Shin, Mi Jin Lee, Jong-Hak Park, Su Jin Kim, Sung Bum Oh, Jonghwan Shin, Seung Min Park, Min Seob Sim, Won Young Kim, In-Cheol Park, Hyun Ho Ryu, Yeonho You, Sang-Chul Kim, Ju Ok Park

**Affiliations:** 1 Department of Emergency Medicine, Chonnam National University Hospital, Gwangju, South Korea; 2 Laboratory of Emergency Medical Services, Seoul National University Hospital Biomedical Research Institute, Seoul, South Korea; 3 Department of Emergency Medicine, Seoul National University College of Medicine and Hospital, Seoul, South Korea; 4 Disaster Medicine Research Center, Seoul National University Medical Research Center, Seoul, Republic of Korea; 5 Department of Emergency Medicine, Yonsei University Wonju College of Medicine, Wonju, South Korea; 6 Yonsei University Wonju Severance Christian Hospital, Wonju-Si, South Korea; 7 Seoul National University Hospital, Seoul, South Korea; 8 Kyungpook National University Hosptial, Daegu, South Korea; 9 Korea University Ansan Hospital, Ansan-Si, South Korea; 10 Dankook University Hospital, Cheonan-Si, South Korea; 11 Seoul National University Boramae Medical Center, Seoul, South Korea; 12 Seoul National University Bundang Hospital, Seongnam, South Korea; 13 Sungkyunkwan University Samsung Medical Center, Seoul, South Korea; 14 Ulsan University Asan Medical Center, Seoul, South Korea; 15 Yonsei University Severance Hospital, Seoul, South Korea; 16 Chonnam National University Hospital, Gwangju, South Korea; 17 Chungnam National University Hospital, Daejeon, South Korea; 18 Chungbuk National University Hospital, Cheongju-Si, South Korea; 19 Hallym University Dongtan Sacred Heart Hospital, Hwaseong-Si, South Korea

**Keywords:** Cardiovascular diseases, Predictive markers, Disease prevention

## Abstract

Genetic, environment, and behaviour factors have a role in causing sudden cardiac arrest (SCA). We aimed to determine the strength of the association between various risk factors and SCA incidence. We conducted a multicentre case-control study at 17 hospitals in Korea from September 2017 to December 2020. The cases included out-of-hospital cardiac arrest aged 19–79 years with presumed cardiac aetiology. Community-based controls were recruited at a 1:1 ratio after matching for age, sex, and urban residence level. Multivariable conditional logistic regression analysis was conducted. Among the 1016 cases and 1731 controls, 948 cases and 948 controls were analysed. A parental history of SCA, low educational level, own heart disease, current smoking, and non-regular exercise were associated with SCA incidence (Adjusted odds ratio [95% confidence interval]: 2.51 [1.48–4.28] for parental history of SCA, 1.37 [1.38–2.25] for low edication level, 3.77 [2.38–5.90] for non-coronary artery heart disease, 4.47 [2.84–7.03] for coronary artery disease, 1.39 [1.08–1.79] for current smoking, and 4.06 [3.29–5.02] for non-regular exercise). Various risk factors related to genetics, environment, and behaviour were independently associated with the incidence of SCA. Establishing individualised SCA prevention strategies in addition to general prevention strategies is warranted.

## Introduction

The incidence of sudden cardiac arrest (SCA) is over 3.7 million annually worldwide^1^, and the annual incidence of SCA is estimated to range from 35 to 55 per 100,000 in the USA and Europe and 28–43 per 100,000 in Asia in the general population^2–4^. Although several studies have been conducted to prevent SCA in the general population over the past few decades, managing it is still challenging^5–8^. SCA is frequently the first clinical manifestation of coronary heart disease (CAD)^9^; however, more than 50% of SCA cases occur without prior knowledge of CAD^10^. Hence, information on the various risk factors involved in SCA incidence would help establish preventive strategies.

Genetic, environmental, and behavioral factors play a role in causing SCA. There is sufficient evidence that parental heart disease, including coronary artery disease (CAD) and SCA, tends to influence the risk of SCA^11–16^. Socioeconomic factors are also associated with the incidence of SCA^17, 18^, and cardiovascular diseases are well-known risk factors for SCA^19, 20^. Moreover, health behaviors such as smoking or exercise have also been reported to be associated with SCA^20–23^. However, it is often difficult to systematically collect data on these various levels of risk factors at the same time. Because individual factors are closely related, the lack of comprehensive information limits understanding of their independent effects, which could contribute to the lack of effective SCA prevention strategies. Furthermore, the association between risk factors and SCA may vary depending on the patient's demographics, such as age and sex; however, studies on this are still lacking.

This study aimed to determine the strength of the association among various risk factors such as a genetic factor (parental history of SCA), socioeconomic factors (including medical aid and educational level), own heart disease, and health behaviors (including smoking and regular exercise) on SCA incidence, and to stratify the analysis into subgroups defined by age and sex.

## Methods

### Study design, setting, and data source

This prospective multicentre case-control study was performed using the project database of the phase II Cardiac Arrest Pursuit Trial with Unique Registration and Epidemiologic Surveillance (CAPTURES-II) in Korea.

The CAPTURES project aimed to identify the risk factors for SCA and evaluate prognosis determinants in a long-term follow-up. The phase I of CAPTURES was conducted in 2014 at 27 hospitals. A detailed description of phase I CAPTURES has been previously reported^24^.

The CAPTURES-II project is a prospective hospital-based patient cohort study conducted at 17 university hospitals in September 2017^20^. From this ongoing project we analysed the data collected until December 2020. This project included patients experiencing out-of-hospital cardiac arrest (OHCA) transported to the emergency department (ED) by the emergency medical service (EMS) with resuscitation efforts and patients with presumed cardiac aetiology identified by emergency physicians in each ED. The CAPTURES-II registry includes face-to-face interviews for patients’ demographics, health behaviors, and comorbidities, medical record review including laboratory tests, short- and long-term follow-up (1-month, 6-month, and 12-month after hospital discharge), and blood sample registration for biomarker evaluations. For the community-based control group, face-to-face interviews and medical records were reviewed to obtain the same SCA and blood sample registration information. Data collected from all participating hospitals were transferred to the Quality Management Committee (QMC), where quality control and statistical analyses were performed. The QMC provided feedback to the study coordinators on the quality management of the data through monthly meetings. When study coordinators could not define a coding element, they consulted emergency physicians in the QMC for clarification. The study protocol was registered at ClinicalTrials.gov (NCT03700203).

### Study population

Adult EMS-treated patients who experienced OHCA with a presumed cardiac aetiology and were transported to 17 participating hospitals from September 2017 to December 2020 were included in the study.

Community-based 1:2 controls were recruited from two university hospitals (one metropolitan and one non-metropolitan). CAPTURES-II control recruitment was promoted in collaboration with public health centres or various community centres, and voluntary applicants were recruited as the control group. Quarterly, a control collection plan was tailored to the characteristics of the case; however, because they were not compared in real-time and there were cases where all planned controls were not gathered, the final data did not exactly match 1:2. In this study, 1:1 control participants who were randomly matched within strata, including age (10-year intervals), sex, and level of urban residence (metropolitan vs. non-metropolitan), were analysed.

### Variable and measurements

The main items were a parental history of SCA (no or yes), socioeconomic factors including medical aid (no or yes), a low educational level (less than or equal to high school) (no or yes), underlying heart disease (no, CAD [myocardial infarction and angina pectoris], non-CAD [heart failure, arrhythmia, structural disease including valvular heart disease, congenital heart disease, and other heart diseases]), health behaviors including current smoking (≥1 cigarette per day within the past month) (no or yes), and non-regular exercise, defined as engaging in moderate-to-vigorous physical activity less than once per week over the past year. The CAPTURES-II project uses the same questionnaire for both the cases and controls^25^. In the case group, after a patient arrives at the ED, physicians at the ED conduct face-to-face interviews with the patients’ families to collect patient information and recruit study participants for the community-based control group. Information about patients’ demographics, socioeconomic factors, health behaviors, and comorbidities was collected. Comorbidities were entered as ‘yes’ only when a doctor or clinic diagnosed them, and treatment was also investigated. In addition to own heart diseases, comorbidity information for hypertension, diabetes mellitus, dyslipidaemia, and stroke was also collected.

### Statistical analysis

The demographic findings of the SCA and community-based control groups are described. Continuous variables were compared using the Wilcoxon rank-sum test, and categorical variables were compared using the Chi-square test. For the case-control dataset, conditional logistic regression analysis was conducted to estimate the association of a parental history of SCA, medical aid, low educational level, own heart disease, current smoking, and non-regular exercise with OHCA incidence and to calculate the adjusted odds ratios (AORs) and 95% confidence intervals (CIs) after adjusting for potential confounders, including hypertension, diabetes mellitus, dyslipidaemia, and stroke. We also conducted a stratified analysis based on age (≥65 years and <65 years) and sex. All statistical analyses were performed using SAS version 9.4 (SAS Institute, Inc., Cary, NC, USA). All p-values were two-tailed, and statistical significance was set at *P* < 0.05.

### Ethics statement

This study was approved by the ethics committees of all 17 participating hospitals (IRB No: Chonnam National University Hospital, CNUH-2017-285; Chungbuk National University Hospital, CBNUH2017-09-009-001; Chungnam National University Hospital, CNUH2017-10-027; Dankook University Hospital, DKUH2018-12-019; Hallym University Kangnam Sacred Heart Hospital, HKS2018-02-016; Hallym University Dongtan Sacred Heart Hospital, HDT2017-10-002; Korea University Anam Hospital, 2018AN0148; Korea University Ansan Hospital, AS17174; Kyungpook National University Hosptial, KNUH2017-10-035-006; Seoul National University Boramae Medical Center, 20171123/30-2017-66/123; Seoul National University Bundang Hospital, B-1711/430-304; Seoul National University Hospital, H-1709-053-883; Soonchunhyang University Bucheon Hospital, SCHBC2018-02-014-002; Sungkyunkwan University Samsung Medical Center, SMC2018-08-121; Ulsan University Asan Medical Center, S2018-1805-0001; Yonsei University Severance Hospital, 4-2017-1201; Yonsei University Wonju Severance Christian Hospital, CR317101). All participants or their proxy provided written informed consent before participating in the study. This study is registered at ClinicalTrials.gov (NCT03700203).

## Results

### Demographic finding

**Figure 1:**
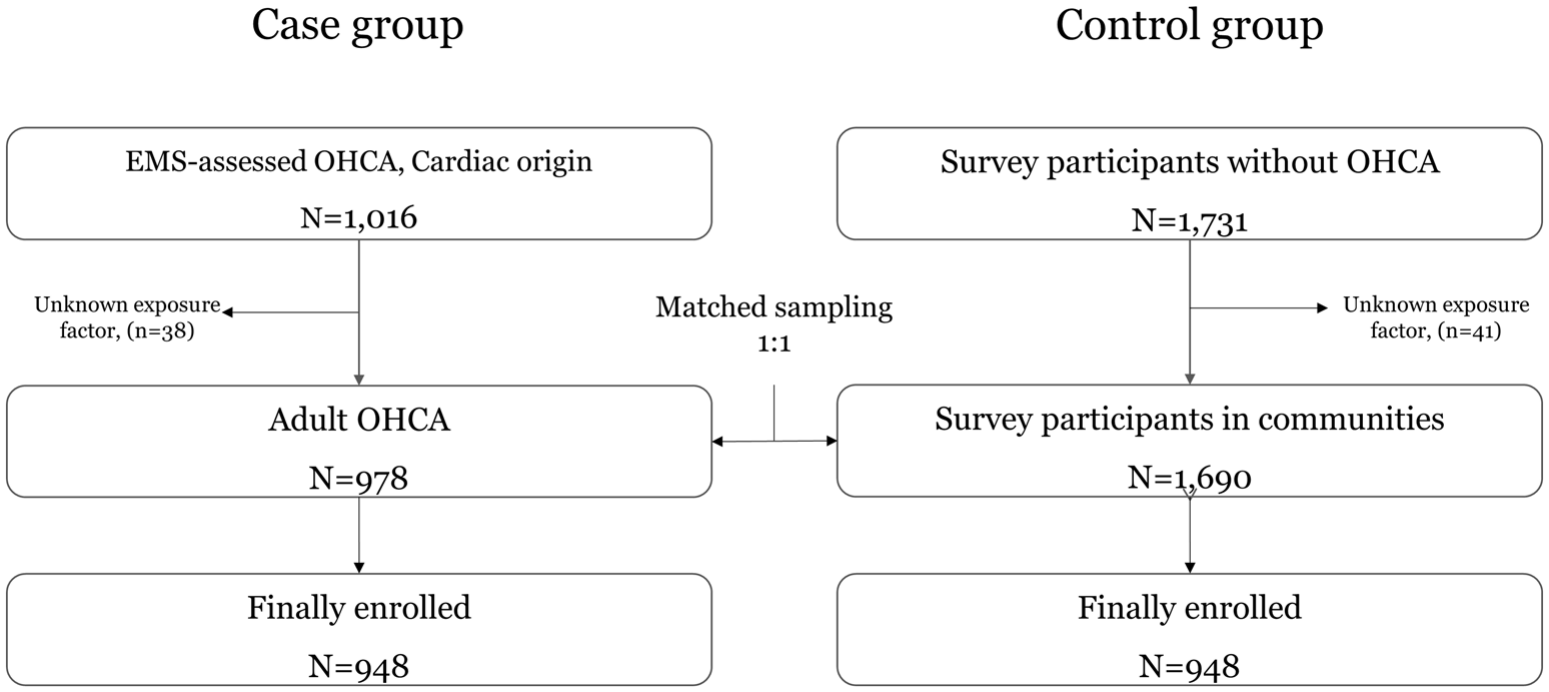
Study population flow.

During the study period, 1016 patients with SCA and 1731 community-based controls were enrolled in the CAPTURES-II project. Among them, by 1:1 matching within strata, age, sex, and urban residence level, 948 SCA cases and 948 community-based matched controls were finally analysed (Fig. 1).

The characteristics of SCA cases and community-based controls are shown in Table 1. Of the cases and matched controls, 64 (6.8%) and 25 (2.6%) had a parental history of SCD; 70 (7.4%) and 39 (4.1%) received medical aid; 705 (74.4%) and 573 (60.4%) had a low educational level; 111 (11.7%) and 31 (3.3%) had a history of CAD; 99 (10.4%) and 30 (3.2%) had non-CAD; 564 (59.5%) and 513 (54.1%) were current smokers; and 685 (72.3%) and 355 (37.4%) were in the non-regular exercise group (all *P* <0.05), respectively.

**Table 1:** Characteristics of the sudden cardiac arrest case group and age and residence level-matched control group.

	Case	Control	*P*
Total	948	948	
Males	688 (72.6%)	688 (72.6%)	1
Age (years)			
19–29	18 (1.9%)	18 (1.9%)	1
30–39	49 (5.2%)	49 (5.2%)	
40–49	135 (14.2%)	135 (14.2%)	
50–59	247 (26.1%)	247 (26.1%)	
60–69	264 (27.8%)	264 (27.8%)	
70–79	235 (24.8%)	235 (24.8%)	
Location of residence, metropolitan	482 (50.8%)	482 (50.8%)	1
Family history			
Parental history of sudden cardiac death	64 (6.8%)	25 (2.6%)	<0.001
Socioeconomic status			
Medical aid	70 (7.4%)	39 (4.1%)	0.002
Low education (≤high school)	705 (74.4%)	573 (60.4%)	<0.01
Heart disease			
No heart disease	738 (77.8%)	887 (93.6%)	<0.001
Non-coronary artery disease	99 (10.4%)	30 (3.2%)	
Coronary artery disease	111 (11.7%)	31 (3.3%)	
Health behaviour			
Current smoking	564 (59.5%)	513 (54.1%)	0.018
Non-regular exercise	685 (72.3%)	355 (37.4%)	<0.001
Other comorbidities			
Diabetes mellitus	252 (26.6%)	133 (14.0%)	<0.001
Hypertension	423 (44.6%)	349 (36.8%)	<0.001
Dyslipidaemia	127 (13.4%)	225 (23.7%)	<0.001
Stroke	78 (8.2%)	28 (3.0%)	<0.001

### Main results

The results of the conditional multivariable logistic regression models, including AORs (95% confidence intervals [CIs] for SCA are shown in Table 2. A parental history of SCA and low educational level were associated with an increased risk of SCA (AOR [95% CI]: 2.51 [1.48–4.28]) for a parental history of SCA; 1.37 [1.38–2.25] for a low educational level. The patient’s own non-CAD and CAD were associated with an increased risk of SCA (AOR [95% CI]: 3.77 [2.38–5.9] for non-CAD and 4.47 [2.84–7.03] for CAD). Regarding health behavior, current smoking and non-regular exercise were associated with increased SCA (AOR [95% CIs]: 1.39 [1.08–1.79] for current smoking and 4.06 [3.29–5.02] for non-regular exercise) (Table 2).

**Table 2:** Risk of parental sudden cardiac death, socioeconomic factors, own heart disease, and health behaviours for sudden cardiac arrest.

	Case/Control	Odds ratio (95% CI)	
		Unadjusted	Adjusted*
Parental history of sudden cardiac death			
No	884/923	Reference	Reference
Yes	64/25	2.69 (1.68–4.31)	2.51 (1.48–4.28)
Medical aid			
No	878/909	Reference	Reference
Yes	70/39	1.88 (1.25–2.82)	1.37 (0.85–2.20)
Low education (≤high school)			
No	243/375	Reference	Reference
Yes	705/573	2.20 (1.77–2.74)	1.76 (1.38–2.25)
Heart disease			
No heart disease	738/887	Reference	Reference
Non-coronary artery disease	99/30	4.42 (2.93–6.68)	3.77 (2.38–5.98)
Coronary artery disease	111/31	4.00 (2.63–6.10)	4.47 (2.84–7.03)
Current smoking			
No	384/435	Reference	Reference
Yes	564/513	1.39 (1.11–1.74)	1.39 (1.08–1.79)
Non-regular exercise			
No	263/593	Reference	Reference
Yes	685/355	4.59 (3.76–5.60)	4.06 (3.29–5.02)

*CI* confidence interval.

*Adjusted for diabetes, hypertension, dyslipidaemia, and stroke.

In the sex-specific multivariable analysis, a parental history of SCA, low educational level, and heart disease were associated with SCA, regardless of sex. However, current smoking was the only risk factor for SCA in women (AOR [95% CI] 8.63 [3.50–21.29]) but not in men (AOR [95% CI] 1.05 [0.80–1.38]) (Table 3).

**Table 3:** Risk of parental sudden cardiac death, socioeconomic factors, own heart disease, and health behaviours for sudden cardiac arrest by sex.

	Male			Female		
	Case/Control	Odds ratio (95% CI)		Case/Control	Odds ratio (95% CI)	
		Unadjusted	Adjusted*		Unadjusted	Adjusted*
Parental history of sudden cardiac death						
No	637/669	Reference	Reference	247/254	Reference	Reference
Yes	51/19	2.84 (1.65–4.86)	2.67 (1.47–4.87)	13/6	2.23 (0.84–5.98)	2.00 (0.61–6.54)
Medical aid						
No	632/656	Reference	Reference	246/253	Reference	Reference
Yes	56/32	1.84 (1.17–2.89)	1.24 (0.74–2.07)	14/7	2.05 (0.82–5.16)	2.34 (0.64–8.52)
Low education (≤high school)						
No	200/301	Reference	Reference	43/74	Reference	Reference
Yes	488/387	2.11 (1.66–2.69)	2.11 (1.66–2.69)	217/186	2.62 (1.58–4.35)	2.04 (1.10–3.78)
Heart disease						
No heart disease	540/640	Reference	Reference	198/247	Reference	Reference
Non-coronary artery disease	64/23	4.06 (2.56–6.46)	3.46 (2.03–5.91)	35/7	5.95 (2.39–14.78)	5.27 (2.06–13.51)
Coronary artery disease	84/25	3.31 (2.03–5.40)	3.82 (2.31–6.33)	27/6	6.43 (2.79–14.82)	7.15 (2.50–20.42)
Current smoking						
No	174/182	Reference	Reference	210/253	Reference	Reference
Yes	514/506	1.06 (0.83–1.36)	1.05 (0.80–1.38)	50/7	8.93 (3.94–20.26)	8.63 (3.50–21.29)
Non-regular exercise						
No	474/232	Reference	Reference	211/123	Reference	Reference
Yes	214/456	4.46 (3.55–5.61)	3.90 (3.06–4.97)	49/137	5.01 (3.34–7.51)	4.98 (3.11–7.98)

*CI* confidence interval.

*Adjusted for diabetes, hypertension, dyslipidaemia, and stroke.

In the age-specific multivariable analysis, a parental history of SCA and heart disease were associated with SCA, regardless of age. However, medical aid, low education, and current smoking were the only risk factors for SCA in the young (AOR [95% CI] 2.45 [1.25–4.82] for medical aid, 2.96 [2.29–3.83] for a low educational level, and 1.59 [1.15–2.18] for current smoking), but not in the old [AOR [95% CI] 0.59 [0.28–1.25] for medical aid, 0.84 [0.51–1.40] for a low educational level, and 1.06 [0.69–1.64] for current smoking]. Non-regular exercise also showed a stronger association with SCA in older adults (AOR [95% CI] 6.36 [4.40–9.20]) than in young individuals (AOR [95% CI] 3.12 [2.39–4.08]) (Table 4).

**Table 4:** Risk of parental sudden cardiac death, socioeconomic factors, own heart disease, and health behaviours for sudden cardiac arrest by age group.

	Young (18–64 y)			Old (>64 y)		
	Case/Control	Odds ratio (95% CI)		Case/Control	Odds ratio (95% CI)	
		Unadjusted	Adjusted^*^		Unadjusted	Adjusted^*^
Parental history of sudden cardiac death						
No	539/554	Reference	Reference	345/369	Reference	Reference
Yes	37/11	3.50 (1.76–6.96)	2.91 (1.35–6.26)	27/14	2.03 (1.05–3.94)	2.22 (1.02–4.84)
Medical aid						
No	526/550	Reference	Reference		Reference	Reference
Yes	50/15	3.64 (2.00–6.62)	2.45 (1.25–4.82)		0.84 (0.46–1.55)	0.59 (0.28–1.25)
Low education (≤high school)						
No	190/323	Reference	Reference		Reference	Reference
Yes	386/242	2.96 (2.29–3.83)	2.96 (2.29–3.83)		0.94 (0.62–1.44)	0.84 (0.51–1.40)
Heart disease						
No heart disease	464/536	Reference	Reference	274/351	Reference	Reference
Non–coronary artery disease	57/16	4.98 (2.68–9.26)	3.56 (1.92–6.61)	42/14	3.99 (2.29–6.95)	3.79 (1.88–7.65)
Coronary artery disease	55/13	4.10 (2.32–7.25)	4.60 (2.34–9.04)	56/18	3.84 (2.05–7.16)	5.27 (2.75–10.12)
Current smoking						
No	205/243	Reference	Reference	179/192	Reference	Reference
Yes	371/322	1.59 (1.20–2.12)	1.59 (1.15–2.18)	193/191	1.10 (0.76–1.59)	1.06 (0.69–1.64)
Non-regular exercise						
No	398/210	Reference	Reference	287/145	Reference	Reference
Yes	178/355	3.83 (2.99–4.92)	3.12 (2.39–4.08)	85/238	6.27 (4.48–8.77)	6.36 (4.40–9.20)

*CI* confidence interval.

*Adjusted for diabetes, hypertension, dyslipidaemia, and stroke.

## Discussion

We found that parental SCA, a low educational level, the patient’s own heart disease, current smoking, and non-regular exercise were independently associated with SCA after adjusting for comorbidities. Additionally, the strength of the association of risk factors for SCA differed according to the patient’s sex and age. Current smoking in women, medical aid and a low educational level in young individuals and non-regular exercise in older adults showed stronger associations with SCA. Our findings suggest that various risk factors related to genetic, environmental, and behavioral factors independently contribute to the incidence of SCA, even if they adjust for each other’s effects. In addition, a general prevention strategy for SCA can be effective; however, individual priorities among risk factors could also vary according to the characteristics of the population.

The finding that a parental history of SCA is a risk factor for SCA is consistent with the findings of other investigations^16, 26^. Previous reports have shown that SCA is more prevalent in individuals with two or more SCAs among first-degree relatives, suggesting that the increased risk of SCA associated with parental SCA may be related to a genetic background^27^. Spooner et al.^28^ suggest that genetic variation in pathologic and physiologic mechanisms may contribute to SCA incidence through three main pathways: (1) atherosclerosis and thrombosis, (2) electrogenesis, and (3) neural regulation and control. However, the underlying genetic mechanisms predisposing individuals to SCA are multifactorial, and detecting essential genetic mutations and polymorphisms can be difficult^27^. Previous studies and our study indicate that a parental history of SCA is independently associated with the risk of OHCA after adjusting for other risk factors. However, there may also be factors related to familial influence, such as hypertension, diabetes, smoking, or physical activity, which increase vulnerability to SCA. In the stratified analysis, we found that parental history of SCD was the only significant risk factor in the male sex group. Moreover, the adjusted odds ratio of a parental history of SCA for SCA was slightly higher in the younger age group than in the older group. A previous study of autopsy results for SCD patients^29^ showed that myocardial hypertrophy, a high genetic predisposition and cause of SCA, was more common in men and younger patients, which may explain our finding. Nonetheless, a definitive explanation cannot be provided.

Socioeconomic factors are also well known risk factors for cardiovascular disease, including SCA. Previous studies also reported a greater disparity in SCA incidence in the younger than in the older age group^17, 18^. We also found that medical aid and a low educational level were significantly associated with SCA only in the young but not in the older age group. The trend of diminishing socioeconomic differences according to age, selective survival in the group with a lower socioeconomic status (i.e. only the healthiest individuals from the lower socioeconomic status group survived until old age), and earlier onset of other diseases or death in the low educational level group could be some possible explanations for our findings^30, 31^.

In our study, patient’s own heart disease, including non-CAD and CAD, showed relatively higher odds for OHCA in the female and younger groups. In general, it was an important risk factor for SCA regardless of age and sex, which is consistent with the results of previous studies^9, 32^.

Similar to previous studies^33, 34^, current smoking status was significantly associated with OHCA in the present study. However, the smoking prevalence was lower in women than in men, and the adjusted odds ratio of current smoking status was only significant in women. A previous study reported that smoking had a higher risk of acute coronary events and cardiovascular death in women than in men^35^. A previous study reported that smoking had a higher risk of acute coronary events and cardiovascular death in women than in men, with findings suggesting that this increased risk in females may be linked to genetic factors related to thrombin signaling^36^. Moreover, the risk of smoking on SCA was only significant in younger patients aged < 65 years, consistent with the findings from a British case-control study, which showed that the association between smoking and myocardial infarction weakened with every 10-year age group^37^. One explanation for this phenomenon could be the ‘depletion of susceptibles’ effect^38^. These results further emphasise the importance of smoking cessation for the female and younger age groups because smoking cessation significantly reduces OHCA risk^33^. Further studies regarding the association of smoking and its cessation with OHCA incidence according to age and sex are needed.

Exercise has a multifactorial effect on OHCA, which occurs more frequently during or shortly after vigorous exercise^39^. However, regular exercise reduces the risk of CAD, including sudden cardiac arrest^39, 40^. Our study also found that regular exercise showed a stronger protective effect against SCA incidence in the age and sex groups. Regular exercise also proved to be a particularly effective effort to reduce OHCA in the group over 60 years of age.

Our study demonstrated the effects of a parental history of SCA, socioeconomic factors, heart disease, and health behavior on SCA incidence, which were previously known to be related to SCA. It also showed that each risk factor had a different effect size according to the sex and age group. Based on these results, risk management according to patient characteristics is required to reduce the burden of SCA in addition to common preventive strategies such as chronic disease management.

Our study had certain limitations. First, our study was a case-control study and not an interventional study. There may have been a significant potential bias that could not be controlled. For example, recall bias could have led to an underestimation or overestimation of the association between risk factors and SCA due to reliance on patient or family member recollections. Selection bias in our control group may have compromised comparability with cases, potentially affecting the strength of our findings. Additionally, misclassification of matching variables could have skewed our risk estimates, altering the apparent magnitude of certain risk factors. In addition, a control group was selected from the same risk population; however, there was a possibility of misclassification of the matching variables. Second, we could not investigate specific information about risk factors such as the cause of parental SCA, diagnosed period of heart disease, smoking period, and intensity of exercise. Third, the historical data on patient risk factors may have been underestimated or overestimated due to reliance on self-reports from patients and guardians during hospital visits. We did not match these self-reports with more objective methods or secondary data sources, such as health insurance records, which could have provided a more balanced and validated dataset. Fourth, in our study, we implemented 1:1 matching using variables such as age, sex, and level of urban residence. However, we were unable to completely overcome issues like selection bias. Fifth, during the data collection phase of our study, we encountered challenges in precisely achieving the planned 1:2 matching ratio based on age, sex, and level of urban residence. Fewer participants than expected were recruited for the control group, which meant that we could not fully meet the optimal comparison framework set out in our research design. Sixth, our study presumed cardiac origin for SCA cases unless a clear non-cardiac origin was evident. This assumption carries the potential for misclassification errors regarding the underlying cause of SCA. Finally, the investigators of our project were not blinded to the study hypotheses, which could have led to biased data collection.

## Conclusion

In our study, a parental history of SCD, low educational level, heart disease, health behavior including smoking, and regular exercise were associated with OHCA incidence. Additionally, each risk factor showed a different effect size according to sex and age. It is necessary to develop a strategy that considers the strength of the association with OHCA risk factors and their different effects according to patient characteristics to establish a prevention program for SCA.

## Data Availability

Datasets were obtained from the Korea Disease Control and Prevention Agency. The datasets generated and/or analysed during the current study are not publicly available.
